# Vigilância e atenção primária à saúde: integração de ações nos municípios rurais remotos brasileiros durante a pandemia da COVID-19

**DOI:** 10.1590/0102-311XPT194324

**Published:** 2025-08-08

**Authors:** Nereide Lucia Martinelli, Simone Schenkman, Lorena Araújo Ribeiro, Paulo Henrique dos Santos Mota, Carinne Magnago, Aylene Emilia Moraes Bousquat

**Affiliations:** 1 Instituto de Saude Coletiva, Universidade Federal de Mato Grosso, Cuiabá, Brasil.; 2 Faculdade de Saúde Pública, Universidade de São Paulo, São Paulo, Brasil.; 3 Universidade Federal de Mato Grosso, Rondonópolis, Brasil.

**Keywords:** Atenção Primária à Saúde, Vigilância em Saúde, COVID-19, Primary Health Care, Public Health Surveillance, COVID-19, Atención Primaria de Salud, Vigilancia en Salud, COVID-19

## Abstract

A crise sanitária decorrente da transmissão comunitária da COVID-19 convocou a vigilância em saúde a exercer papel central no enfrentamento da pandemia. Este estudo analisa as contribuições e dificuldades da vigilância em saúde para desenvolver as ações de enfrentamento à pandemia da COVID-19 em municípios rurais remotos brasileiros na perspectiva dos gestores, profissionais e usuários. Estudo qualitativo de casos múltiplos com análise temática e dedutiva de 51 entrevistas, conduzidas com gestores e profissionais de saúde em 16 municípios rurais remotos dos estados de Rondônia, Mato Grosso, Tocantins, Piauí, Minas Gerais e Amazonas, e 29 entrevistas de usuários, agentes comunitários de saúde e movimentos sociais de oito municípios rurais remotos. Durante a pandemia da COVID-19, enfrentaram dificuldades para operacionalizar a vigilância em saúde e o acesso dos sintomáticos respiratórios aos serviços de saúde. Necessitaram reorganizar a rede de atenção à saúde, obter cooperação técnica, realizar a governança intergestores e buscar alternativas para operacionalizar o sistema locorregional de saúde. Os principais obstáculos enfrentados no cuidado foram relativos à articulação interfederativa, insuficientes para fortalecer a rede assistencial, laboratorial e o sistema logístico. Os condicionantes políticos, estruturais e organizacionais confirmaram que os gestores locais contribuíram para conter os riscos, e se empenharam para: estabelecer arranjos e relações intersetoriais locais, integrar a vigilância em saúde à atenção primária à saúde, reorganizar os processos de trabalho e a comunicação com a população. A superação efetiva e sustentável das dificuldades enfrentadas pelos municípios rurais remotos requer compartilhamento interfederativo pautado pelos princípios da integralidade, intersetorialidade e participação social.

## Abstract (en)

The health crisis resulting from community transmission of COVID-19 called for the health surveillance to play a central role in facing the pandemic. This study analyzes the contributions and difficulties faced by health surveillance in the development of actions to combat the COVID-19 pandemic in Brazilian remote rural municipalities from the perspective of managers, professionals and users. This was a qualitative study of multiple cases that involved a thematic and deductive analysis of 51 interviews conducted with managers and health professionals in 16 remote rural municipalities in the states of Rondônia, Mato Grosso, Tocantins, Piauí, Minas Gerais, and Amazonas, and 29 interviews with users, community health agents and social movements in eight remote rural municipalities. During the COVID-19 pandemic, they faced difficulties in operationalizing health surveillance and ensuring the access of patients with compromised respiratory status to health services. They needed to reorganize the health care network, establish technical cooperation, conduct inter-agency governance and seek alternatives to operationalize the local health system. The main obstacles faced in care were related to articulation between states, which were insufficient to strengthen the care and laboratory network and the logistical system. The political, structural and organizational constraints confirmed that local managers contributed to contain the risks and made an effort to establish local intersectoral arrangements and relations, integrate health surveillance with primary health care, reorganize work processes and communication with the population. Effectively and sustainably overcoming the difficulties faced by remote rural municipalities requires cooperation between states guided by the principles of comprehensiveness, intersectoriality, and social participation.

## Resumen

La crisis sanitaria derivada de la transmisión comunitaria de la COVID-19 requirió que la vigilancia en salud asumiera un papel central en el enfrentamiento de la pandemia. Este estudio analiza las contribuciones y dificultades de la vigilancia en salud para desarrollar acciones contra la pandemia de COVID-19 en municipios rurales remotos brasileños desde la perspectiva de los gestores, los profesionales y los usuarios. Se trata un estudio cualitativo de múltiples casos con análisis temático y deductivo de 51 entrevistas, realizadas a gestores y profesionales de salud en 16 municipios rurales remotos de los estados de Rondônia, Mato Grosso, Tocantins, Piauí, Minas Gerais y Amazonas, y 29 entrevistas con usuarios, agentes comunitarios de salud y movimientos sociales de ocho municipios rurales remotos. Durante la pandemia de COVID-19, se enfrentaron numerosas dificultades para las operaciones de la vigilancia en salud y el acceso de las personas con síntomas respiratorios a los servicios de salud. Fue necesario reorganizar la red de atención sanitaria, obtener cooperación técnica, realizar gobernanza entre los gestores y buscar alternativas para operar el sistema sanitario locorregional. Los principales obstáculos a los que se enfrentó la atención sanitaria se relacionaron con la articulación interfederativa, insuficiente para fortalecer la red asistencial, los laboratorios y el sistema logístico. Los condicionantes políticos, estructurales y organizativos confirmaron que los gestores locales contribuyeron a contener los riesgos y se esforzaron por establecer acuerdos y relaciones intersectoriales locales, integrar la vigilancia en salud en la atención primaria de salud, reorganizar los procesos de trabajo y la comunicación con la población. La superación efectiva y sostenible de las dificultades a las que se enfrentan los municipios rurales remotos requiere un intercambio interfederativo basado en los principios de integralidad, intersectorialidad y participación social.

## Introdução

A Emergência em Saúde Pública de Importância Nacional ^1^, em decorrência da transmissão comunitária da COVID-19, exigiu que a vigilância em saúde exercesse papel central no enfrentamento da crise sanitária. O Plano de Contingência Nacional da COVID-19 ^2^ recomendou aos estados e municípios a elaboração dos seus próprios planos, e a reorganização da rede de atenção locorregional ^3^ ^,^ ^4^ ^,^ ^5^. A vigilância em saúde, de forma integrada à atenção primária à saúde (APS), foi responsável por implementar medidas de contenção, prevenção, detecção, controle e monitoramento dos casos e contatos, além de disseminar informações contingenciais nos territórios ^1^ ^,^ ^2^ ^,^ ^6^ ^,^ ^7^ ^,^ ^8^.

Em meio a um cenário de incertezas e resistências no enfrentamento da pandemia da COVID-19, tanto por parte dos entes federativos quanto pela população, a função essencial da vigilância em saúde no Sistema Único de Saúde (SUS) ganha uma centralidade ainda mais relevante. Recorda-se que a vigilância em saúde deve abranger todos os níveis e formas de atenção à saúde, orientar o modelo de atenção e atuar com base no conhecimento do território. Com a participação da comunidade, e articulada a outros setores, deve expandir sua atuação sobre os determinantes e condicionantes da saúde visando à integralidade e equidade no cuidado ^9^. De forma ativa, a vigilância em saúde atua na promoção da saúde, com atividades educativas, busca ativa, notificação, acompanhamento e monitoramento dos casos. De forma passiva, contribui para elaborar relatórios, analisar dados e compartilhar informações institucionais para monitorar fatores de risco, realizar rastreamento e comunicar-se com as pessoas e instituições ^6^.

Na vigilância em saúde, a descentralização exigiu responsabilidade interfederativa e mecanismos para a governança, permitiu aproximação da gestão das ações à realidade local e favoreceu a transferência de recursos financeiros para o enfrentamento das desigualdades sociais no território nacional. No entanto, as fragilidades da rede de serviços municipais e regionais afetam sua capacidade de resposta, especialmente em situações que constituem emergências sanitárias, como foi o caso da COVID-19 ^6^ ^,^ ^10^ ^,^ ^11^ ^,^ ^12^. Fragilidades que precisam ser consideradas para garantir a eficácia do sistema municipal e integrar a vigilância em saúde à APS, reduzir as desigualdades no acesso à saúde e promover uma gestão mais coordenada para os cuidados de saúde ^13^ ^,^ ^14^ ^,^ ^15^.

Na pandemia da COVID-19, os desafios para reestruturar os sistemas e serviços de saúde ocorreram em todo o mundo. No Brasil, tornaram-se tarefas complexas, considerando que 60,4% dos municípios são classificados como rurais, compondo 16,9% da população total ^16^. Destes, 54,6% são rurais adjacentes e 5,8% são rurais remotos (n = 323). Nesses municípios, há desigualdades socioespaciais e imensas dificuldades, destacando-se a baixa capacidade instalada na rede de atenção à saúde, a distância dos centros de referência e os problemas logísticos para acessar os serviços de saúde ^12^ ^,^ ^17^ ^,^ ^18^ ^,^ ^19^.

Estudos mostram que as características dos municípios rurais remotos condicionam a provisão da APS ^5^ ^,^ ^12^. Durante a pandemia, a APS foi a principal porta de entrada para o sistema de saúde, mas o acesso agravou-se pela escassez de serviços e profissionais, especialmente médicos. Nestes, as dificuldades de acesso pré-pandemia foram intensificadas, e a vigilância e o cuidado dos sintomáticos respiratórios podem ter sido influenciadas pelas desigualdades socioespaciais ^12^ ^,^ ^19^, insuficiência de leitos hospitalares e de UTI, falta de exames bioquímicos e de imagem, entre outros. Além disso, a distância até os grandes centros, a insuficiência de transporte e a dependência das transferências intergovernamentais impactaram no enfrentamento ^5^ ^,^ ^12^ ^,^ ^17^ ^,^ ^20^.

O objetivo deste estudo é discutir as contribuições e as dificuldades da vigilância em saúde para desenvolver as ações de enfrentamento da pandemia da COVID-19 em municípios rurais remotos, especialmente na perspectiva dos gestores, profissionais e usuários.

## Método

Estudo de casos múltiplos que faz parte da pesquisa nacional *Desafios e Estratégias no Enfrentamento da Pandemia da COVID-19 nos Municípios Rurais e Remotos Brasileiros*. Utiliza a abordagem qualitativa ^21^ e o referencial de políticas públicas ^22^ para analisar as ações desenvolvidas nos municípios rurais remotos durante a pandemia da COVID-19. Realiza análise multidimensional ^23^ dos condicionantes políticos, estruturais e organizacionais, identifica os segmentos envolvidos, a tomada de decisões e as contribuições na aplicação das medidas de contingenciamento do SARS-CoV-2, para entender como as ações de vigilância em saúde foram conduzidas frente às necessidades que emergiram nos municípios rurais remotos.

**Figura 1:**
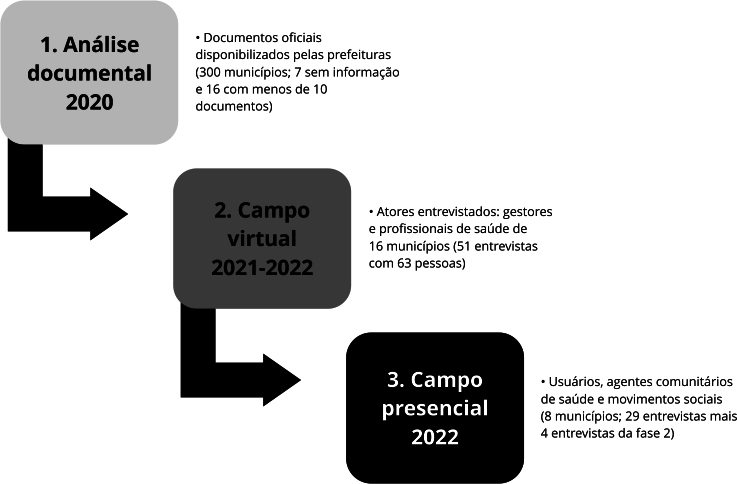
Etapas da pesquisa nacional que embasaram este estudo sobre o enfrentamento da pandemia de COVID-19 nos municípios rurais remotos brasileiros (2020-2022).

O estudo foi desenvolvido em três etapas subsequentes (Figura 1). Na primeira, em 2020, foi realizada a busca e análise dos planos de contingência municipais e outros documentos oficiais (decretos, boletins e informes) disponibilizados pelas prefeituras dos 323 municípios rurais remotos. Optou-se por excluir da análise documental aqueles municípios rurais remotos em que menos de dez documentos tivessem sido encontrados nos sites e/ou mídias sociais oficiais do município. Assim, 300 municípios mantiveram-se no estudo. Com base na análise documental e como critério de seleção, foi criado um escore de enfrentamento (0 a 1), orientado pelas dimensões: Condução da política; Apoio social; Organização dos serviços; Vigilância de casos; Isolamento social; e Vigilância de fronteiras. Entre os municípios rurais remotos com os escores de maior e menor completude, 16 foram selecionados para a realização das entrevistas.

Fonte: elaboração própria.

Na segunda etapa, entre julho de 2021 e abril de 2022, atores da gestão municipal e da prestação de serviços dos 16 municípios rurais remotos foram entrevistados virtualmente: secretários municipais de saúde (SMS); coordenadores da atenção básica (AB) e/ou vigilância em saúde (VS) e profissionais da Estratégia Saúde da Família (PESF); prefeitos (Gestor); representantes do Conselho Municipal de Saúde (CMS); responsáveis pela regulação; e assistentes sociais (AS). As entrevistas (n = 51) com 63 atores foram gravadas por videoconferência no Google Meet (https://meet.google.com) e da plataforma Zoom (https://zoom.us/).

Na terceira etapa, em 2022, no campo presencial, foram entrevistados os usuários adoecidos pelo SARS-CoV-2 (Usu) e agentes comunitários de saúde (ACS). As entrevistas (n = 29) foram gravadas e realizadas nos municípios de Goianorte (Tocantins), Indaiabira e Rubelita (Minas Gerais), Maués (Amazonas), Nova Lacerda e Vila Bela da Santíssima Trindade (Mato Grosso), e Pajeú do Piauí e Porto Alegre do Piauí (Piauí) (Tabela 1). Estes foram escolhidos em função de visitas anteriores, acessibilidade e os modos de enfrentamento da pandemia.

**Tabela 1:** Municípios rurais remotos selecionados segundo informações sociodemográficas. Brasil, 2019.

Município rural remoto	UF	População *	Área (km^2^) **	Densidade demográfica **	PIB *per capita* (R$) **	Bolsa Família (% da população) ***	IDHM ^#^	Índice de Gini ^#^
São Miguel do Guaporé	Rondônia	23.005	7.815,0	2,99	16.701	14,05	Médio	0,63
Maués ^##^	Amazonas	63.905	39.988,0	1,50	6.272	54,67	Baixo	0,64
Indaiabira ^##^	Minas Gerais	7.351	1.004,1	7,50	6.650	46,73	Médio	0,48
Rubelita ^##^	Minas Gerais	5.995	1.110,2	6,39	5.598	48,63	Baixo	0,50
Pajeú do Piauí ^##^	Piauí	3.389	1.075,0	3,07	5.059	62,08	Baixo	0,59
Porto Alegre do Piauí ^##^	Piauí	2.710	1.137,0	2,26	7.853	54,70	Baixo	0,48
Campos Lindos	Tocantins	10.116	3.240,0	2,90	31.756	26,52	Baixo	0,68
Goianorte ^##^	Tocantins	5.123	1.801,0	2,85	9.093	56,38	Médio	0,54
Campinápolis	Mato Grosso	15.980	5.978,9	2,59	10.525	24,71	Baixo	0,69
Figueirópolis D’Oeste	Mato Grosso	3.494	891.4	3,95	13.443	8,09	Médio	0,43
Indiavaí	Mato Grosso	2.752	592,5	4,22	13.307	14,29	Médio	0,48
Nova Canaã do Norte	Mato Grosso	12.787	5.953,1	2,07	25.332	10,02	Médio	0,57
Nova Lacerda ^##^	Mato Grosso	6.640	4.780,4	1,28	20.447	29,10	Médio	0,53
Serra Nova Dourada	Mato Grosso	1.650	1.490,7	1.01	12.359	44,58	Médio	0,46
Terra Nova do Norte	Mato Grosso	9.667	2.399,7	3,74	16.661	20,11	Médio	0,50
Vila Bela da Santísssima Trindade ^##^	Mato Grosso	16.128	13.443,6	1,14	21.813	22,06	Médio	0,59

IDHM: Índice de Desenvolvimento Humano Municipal; PIB: produto interno bruto; UF: Unidade Federativa.

* De acordo com dados do Instituto Brasileiro de Geografia e Estatística ^37^;

** De acordo com dados do Instituto Brasileiro de Geografia e Estatística ^38^;

*** De acordo com dados do Ministério do Desenvolvimento e Assistência Social, Família e Combate à Fome ^39^;

^#^ De acordo com dados do Programa das Nações Unidas para o Desenvolvimento ^40^;

^#^ ^#^ Município rural remoto com campo presencial, além do virtual.

Para as entrevistas, roteiros semiestruturados foram elaborados com base no instrumental de análise de políticas públicas ^22^ e da categoria acesso como referencial teórico ^24^, e ajustados a cada segmento para levantar informações sobre os condicionantes do enfrentamento da pandemia e respectivas categorias de análise ^24^ (Quadro 1). As categorias de análise foram pré-definidas, de modo a refletir os referenciais teóricos, mas também permitir categorias emergentes a partir das respostas ao roteiro. De forma transversal a esses condicionantes, foram analisadas a gestão e a condução da pandemia no município e região, na percepção dos usuários que vivenciaram o adoecimento pela COVID-19.

**Quadro 1:** Condicionantes e categorias de análise das entrevistas.

CONDICIONANTES	CATEGORIAS DE ANÁLISE
Político	(a) Plano de contingência (medidas protetivas e sua comunicação): suspensão das atividades, distanciamento social, barreiras sanitárias, rondas, isolamento social, quarentena; e vigilância de casos-identificação e monitoramento de casos e de contactantes; (b) Comitê de Enfrentamento: participação dos coordenadores de atenção básica e vigilância em saúde com outros setores públicos/privados/sociedade em relação à instituição das medidas protetivas; (c) Decretos: implementação e fiscalização das medidas protetivas; (d) Intersetorialidade no trabalho de contingenciamento.
Estrutural	(a) Mudança estrutural e adaptações na estrutura física existente; (b) Capacidade instalada laboratorial; (c) Aquisição dos insumos específicos e equipamentos de apoio laboratorial e proteção individual (máscaras, sanitizantes, oxímetros, O_2_, termômetro; (d) RT-PCR e tomografia computadorizada (vigilância de casos); (e) Disponibilidade de testes rápidos; (f) Vacinação - rede de frio; (g) Equipe de vigilância (disponibilidade, contratação e capacitação); (h) Disponibilidade de transporte, envio de amostras laboratoriais e encaminhamentos dos usuários com sintomas respiratórios; (i) Estrutura de tecnologia da informação e comunicação (disponibilidade de telefone fixo e celular, conectividade; sistema de informações e monitoramento de casos).
Organizacional	(a) Acompanhamento e atendimento presencial e remoto; (b) Separação do acesso para atendimentos de rotina e de sintomáticos respiratórios; (c) Definição de equipes específicas para acompanhamento dos sintomáticos (presencial e remoto); (d) Realocação de profissionais na equipe de vigilância; (e) Ampliação das atribuições dos profissionais da atenção primária à saúde para a vigilância em saúde; (f) Disponibilidade de protocolos organizacionais; (g) Fluxos e reorganização dos serviços de atenção à saúde para diagnóstico e monitoramento; (h) Fluxos e reorganização dos serviços de apoio laboratorial local e regional; (i) Monitoramento dos sinais clínicos dos sintomáticos em isolamento domiciliar (visita peridomiciliar e remoto); (j) Notificação de casos pelas equipes; (l) Busca ativa de sintomáticos e de contatos; (m) Comunicação de risco e educação em saúde.

RT-PCR: reação em cadeia da polimerase com transcrição reversa.

Os roteiros semiestruturados, considerando todos os segmentos, foram utilizados para a elaboração de um instrumento guia para a extração de informações e para a análise do conteúdo das entrevistas. Esse instrumento foi testado por duas pesquisadoras, e, após ajustes, novos testes foram realizados com o conjunto de pesquisadores. Após conclusão da análise do conjunto das entrevistas, foi realizada uma síntese global de cada município, destacando de forma combinada as falas mais representativas.

A análise das entrevistas presenciais considerou as categorias pré-definidas ^24^ (Quadro 1) e relacionou as medidas protetivas da vigilância em saúde à disponibilidade de serviços e de profissionais para identificação, confirmação, isolamento e acompanhamento dos casos; às distâncias a serem vencidas para a testagem e exames para a confirmação de casos (tomografia computadorizada - TC); à vacinação; e à acessibilidade financeira.

Os entrevistados foram identificados pelas siglas dos atores, seguidas por dois numerais separados por ponto, sendo um representativo de cada um dos 16 municípios rurais remotos e outro indicativo do entrevistado (p.ex.: Usu15.6).

Os entrevistados, mediante concordância, assinaram o Termo de Consentimento Livre e Esclarecido. A pesquisa foi aprovada pelo Comitê de Ética da Faculdade de Saúde Pública da Universidade de São Paulo (CAAE 37672620.8.0000.5421; parecer nº 4.285.824).

## Resultados

Os 16 municípios rurais remotos selecionados para a etapa II estão localizados nos estados do Amazonas, Minas Gerais, Piauí, Tocantins, Mato Grosso e Rondônia. Os municípios rurais remotos dos quatro primeiros estados apresentam baixa densidade demográfica e população beneficiada pelo Bolsa Família acima de 45%. Em sua maioria (n = 10), o Índice de Desenvolvimento Humano Municipal (IDHM) é de médio desenvolvimento (Tabela 1). São municípios de pequeno porte, onde a rede de atenção à saúde é constituída basicamente por serviços de APS. Apenas três deles possuem hospital, um tem unidade mista de saúde e os demais encaminham suas demandas para os serviços de referência da região de saúde.

### Condução política

Seguindo os decretos dos governos federal e estadual, os 16 municípios rurais remotos criaram o Comitê de Contingenciamento da COVID-19, reorganizaram e conduziram as ações para integrar a vigilância em saúde à APS. De caráter consultivo, e diferenciados, os comitês foram constituídos por representantes do governo e da população: prefeitos; servidores das secretarias de saúde, educação e promoção/assistência social; polícia municipal e promotoria pública; Conselho Municipal de Saúde; comércio; e religiosos.

Em alguns municípios rurais remotos, as medidas de contingenciamento foram elaboradas antes da ocorrência do primeiro caso e afixadas em pontos estratégicos para, segundo entrevistado, “*conhecimento, lembrete de uso da máscara, álcool gel*” (CMS4), e divulgadas pelas emissoras de rádio, “*redes sociais, carros de som*” (CMS4).

Com variações entre os municípios rurais remotos, decidiu-se por implementar barreiras e rondas sanitárias; toques de recolher; isolamento e distanciamento social; suspensão dos serviços essenciais e não essenciais; proibição de aglomerações e festas; suspensão dos espaços de lazer; e sanitização dos veículos na vigilância de fronteiras. Nem todos realizaram a vigilância de fronteiras, por falta de profissionais.

As ações de vigilância em saúde foram realizadas pelos profissionais das SMS (médicos, enfermeiros, fisioterapeutas, psicólogos, cirurgiões dentistas, agentes de vigilância ambiental, agentes da vigilância sanitária e ACS), secretarias de educação (professores), obras (motoristas), assistência social (assistente social) e da Polícia Militar. Alguns profissionais foram realocados ou contratados, considerando o déficit existente antes da pandemia. Para as condições mínimas de biossegurança dos profissionais, foram oferecidos equipamentos de proteção individual (EPIs).

Antes da pandemia, alguns municípios rurais remotos tinham unidades de vigilância em saúde registradas no Departamento de Informática do SUS (DATASUS), ou seja, estabelecimentos isolados que realizam trabalho de campo a partir de casos notificados (Tabela 2). No pós-pandemia, Rondônia instalou novas unidades de Serviço de Apoio Diagnóstico e Terapêutico (SADT) e farmácias, enquanto Mato Grosso implantou laboratórios de saúde pública.

**Tabela 2:** Municípios rurais remotos, segundo Unidade Federativa (UF), região de saúde e rede de atenção laboratorial. Brasil, 2019/2022.

Município rural remoto	UF	Rede local laboratorial							
		SADT isolado		Farmácia		Laboratório de saúde pública		Unidade de vigilância em saúde	
		2019	2022	2019	2022	2019	2022	2019	2022
São Miguel do Guaporé	Rondônia	3	6	1	10	-	-	2	2
Maués	Amazonas	1	1	1	1	-	-	1	1
Indaiabira	Minas Gerais	1	-	-	1	-	-	1	1
Rubelita	Minas Gerais	1	1	1	1	-	-	1	1
Pajeú do Piauí	Piauí	1	-	1	1	-	-	-	-
Porto Alegre do Piauí	Piauí	-	-	1	1	-	-	-	-
Campos Lindos	Tocantins	-	1	1	1	-	-	-	1
Goianorte	Tocantins	1	-	1	1	-	-	1	1
Campinápolis	Mato Grosso	-	-	1	1	-	-	-	-
Figueirópolis D’Oeste	Mato Grosso	-	-	1	1	1	1	-	-
Indiavaí	Mato Grosso	1	1	1	1	-	-	-	-
Nova Canaã do Norte	Mato Grosso	3	3	2	2	1	1	-	-
Nova Lacerda	Mato Grosso	1	1	1	1	2	2	-	-
Serra Nova Dourada	Mato Grosso	-	-	1	1	-	1	-	-
Terra Nova do Norte	Mato Grosso	2	2	-	-	1	1	-	-
Vila Bela da Santísssima Trindade	Mato Grosso	2	2	2	1	-	1	1	-

SADT: Serviço de Apoio Diagnóstico e Terapêutico.

Fonte: elaboração própria com base nos dados do Instituto Brasileiro de Geografia e Estatística ^41^ e do Departamento de Informática do SUS ^42^.

As reuniões da Comissão Intergestores Regional e a cooperação técnica das instâncias regionais das Secretarias Estaduais de Saúde (SES) incentivaram o compartilhamento das experiências, “*tudo por live; faziam sugestões e o que era possível, no município, era adotado*” (PESF/AB3). Afirmaram que reorganizar a vigilância em saúde “*foi difícil*” (AB3) e que seguiram os protocolos e as notas técnicas, mas tiveram “*muitas dúvidas e orientar o paciente era muito difícil*” (AS8). Para eles, foi um processo “*abrupto, a gente, ficou meio perdido*” (AB3).

Os comitês de contingenciamento decidiram antecipar as medidas de vigilância em saúde para orientar, acolher, atender, tratar a população e garantir a segurança dos profissionais. Estes realizaram atendimento de sintomáticos, triagem, coleta de materiais para exames RT-PCR, testes rápidos, notificação, investigação e observação de casos. Usuários em isolamento foram monitorados virtualmente e receberam visitas peridomiciliares.

### Estrutura

No início da pandemia da COVID-19, alguns estabelecimentos de saúde da rede de atenção local foram fechados. Posteriormente, criaram centros de COVID-19 ou definiram locais específicos para o atendimento às pessoas com sintomas respiratórios. A rede de apoio laboratorial (Tabela 2), inexistente na maioria dos municípios rurais remotos, e a falta de testes rápidos dificultaram a detecção, a confirmação dos casos e o tratamento. Alguns municípios rurais remotos abriram “*laboratórios para fazer exames mais básicos*” (SMS10), outros estabeleceram salas de coleta. Em ambos, o fluxo de atendimento foi reorganizado, com horários específicos, mas a oferta não supria as necessidades.

Durante a pandemia, apesar do auxílio de alguns Consórcios Intermunicipais de Saúde, a baixa complexidade da rede local e a insuficiência das cotas da Programação Pactuada e Integrada (PPI) resultaram em deslocamentos diários dos residentes dos 16 municípios rurais remotos para os serviços de referência nas regiões de saúde e ou para as capitais. A maioria relatou insuficiência de transporte sanitário adequado para encaminhar as amostras de RT-PCR (reação em cadeia da polimerase com transcrição reversa) aos Laboratórios Centrais (LACENs), insuficiência de vagas hospitalares e dificuldade de acesso aos serviços para a confirmação diagnóstica por TC. A falta de profissionais capacitados comprometeu o lançamento de informações no Sistema Gerenciador de Ambiente Laboratorial (GAL), sistema nacional que integra as redes estaduais de laboratórios de Saúde Pública.

Os preços elevados e a escassez dos equipamentos de apoio laboratorial e EPIs no início da pandemia comprometeram sua aquisição e entrega. Os entrevistados afirmaram receber recursos financeiros do Governo Federal em suficiência e no tempo adequado. As prefeituras também disponibilizaram recursos financeiros para compra de EPIs, mas “*não tinha onde comprar*” (PESF5); “*faltou a máscara, mas sempre conseguia de município vizinho*” (PESF5). Essas situações foram parcialmente contornadas por doações de EPIs e álcool em gel pela comunidade, instituições e empresas locais. Alguns gestores mandaram “*confeccionar máscaras de tecido*” (SMS15) e distribuir para a população.

Alguns municípios rurais remotos adquiriram oxímetros e termômetros. O oxigênio não faltou, mas houve dificuldades para manter os estoques, concentrados nos grandes centros e capitais. Uma exceção foi Maués, que tinha produção local antes da pandemia.

Para armazenar as vacinas de COVID-19, a rede de frio em alguns municípios rurais remotos passou por readequações: “*não temos câmara fria*” (PESF14); em outros, “*supriu a necessidade. A central de imunização recebe, distribui e controla*” (AB10).

A vigilância em saúde integrada à APS permitiu a formação de grupos de trabalho, denominados pelos entrevistados como “*equipes de vigilância em saúde*”. Alguns municípios rurais remotos tiveram dificuldades para contratar profissionais, como informa um entrevistado: “*teria que ter contratado mais, mas não tinha gente para trabalhar*” (VS7); e a capacitação ocorreu pelo uso dos protocolos e notas técnicas, com as quais “*foram aprendendo, ia repassando, foi o que deu para fazer*” (SMS2). Outros definiram horário protegido: “*quarta-feira parávamos para qualificar*” (ESF3).

Durante a pandemia, alguns municípios rurais remotos ampliaram a capacidade e a cobertura de internet, facilitando a comunicação com a população. A estrutura de serviços foi utilizada para monitorar os casos, realizar atividades educativas, organizar as barreiras e rondas sanitárias. Foram utilizados telefones institucionais, mas também dispositivos de uso pessoal: “*deixamos à disposição o telefone da gente 24 horas por dia*” (PESF/AB3). Boletins, panfletos e cartazes foram elaborados e as informações divulgadas por meio das redes sociais, carros de som e emissoras de rádio. Segundo os entrevistados, tanto no perímetro urbano quanto nas comunidades rurais a comunicação foi dificultada pela insuficiência de veículos.

### Organização

As ações de vigilância em saúde foram realizadas pelos profissionais “*médico, enfermeiro*” (PESF/AB3) e ACS, sendo organizadas para o atendimento presencial e remoto, busca ativa de sintomáticos e de contactantes. Segundo entrevistados, os sintomáticos respiratórios “*não iam procurar, a gente fez campanha orientando a procurar o centro de COVID*” (SMS2). Nas áreas rurais, os ACS monitoraram os casos e orientaram a população com apoio dos profissionais da vigilância em saúde, que continuaram se deslocando até “*as comunidades rurais*” (SMS10) para orientar e realizar o teste rápido.

Ao comunicar o risco, a população foi orientada quanto “*à gravidade do problema*” (AB9). As equipes elaboraram “*panfletos, cartazes, conversaram com lideranças comunitárias*” (AB10), “*todos se engajaram*” (PESF8). O prefeito (médico) de um dos municípios rurais remotos gravou vídeos, “*as pessoas esperavam todos os dias às oito horas da noite a publicação do doutor*” (PESF8). Um dos entrevistados referiu: “*nunca acreditei que isso funcionasse, as redes sociais*” (PESF8).

Durante o isolamento de “*quatorze dias*” (AB10) e adoecimento, o monitoramento dos casos foi priorizado pelas visitas peridomiciliares e contato por telefone: “*ia de casa em casa*” (PESF14) e “*fazia ligação, pacientes e contactantes*” (SMS10) ou por WhatsApp. Alguns municípios rurais remotos “*deixavam o oxímetro*” (PESF/AB3) nas residências, outros disponibilizavam o termômetro. O monitoramento aumentou o elo entre a população e profissionais da APS/vigilância em saúde; tal foi o ponto de integração, que as equipes se mesclavam no atendimento dos usuários e no monitoramento da situação pandêmica.

De forma distinta, a vigilância em saúde foi organizada para prevenir a disseminação do vírus. Foram estabelecidos fluxos e horários de atendimento diferenciados: “*agendavam meu horário, cada um isolado*” (Usu14.1). A triagem dos sintomáticos ocorria do lado de fora da unidade básica de saúde (UBS) ou em áreas abertas de escolas. Os profissionais seguiram o fluxo de atendimento e tratamento organizado pelo municípios rurais remotos: “*o tratamento não pode ser igual para todo mundo, fizemos um protocolo*” (PESF7.2).

A falta de capacitação no Sistema de Informação em Saúde para a Atenção Básica (e-SUS) foi um desafio para alguns municípios rurais remotos: “*nunca tinha trabalhado com o e-SUS, não estávamos conseguindo digitar as notificações de COVID. No sistema constava vinte, trinta casos, e já estávamos com trezentos*” (PESF9).

Para realizar a assistência e as ações de vigilância em saúde nos municípios rurais remotos, os profissionais foram realocados, conforme as necessidades, “*na barreira, disque corona, monitoramento, digitação de medicações*”, “*o pessoal vestiu a camisa*” (PESF9). A realocação dos profissionais integrou as atividades e os setores, mas os entrevistados destacaram preocupações para o período pós-crise: “*a atenção básica não tem mais vínculo, eu nem sei as pessoas da minha área que estão com COVID*” (PESF9).

Onze municípios rurais remotos organizaram barreiras sanitárias terrestres e fluviais nas entradas da cidade, nos pontos turísticos e áreas de lazer. Os “*agentes de saúde, fiscais sanitários, agentes de endemias, bombeiros, polícia militar, polícia civil*” (VS8) e outros profissionais participaram ativamente. Eles monitoravam “*quem entrava e saía do município*” (AB13), e agiam caso o “*doente tentasse entrar sem se identificar*” (SMS10). Além disso, realizavam sanitização de veículos, orientações, cadastravam e identificavam os sintomáticos, aferiam a temperatura e encaminhavam para consulta ou isolamento.

Alguns municípios rurais remotos não instalaram as barreiras sanitárias em razão da falta de testes rápidos e da escassez de profissionais. Houve críticas direcionadas a um gestor específico que não a instalou: “*deixou a desejar. Aqui tem um fluxo muito grande, as pessoas vêm para ranchos aos finais de semana* [pescaria]” (PESF/AB3). Outros municípios criaram equipes ambulantes como alternativa para monitorar e prestar assistência às comunidades.

A suspensão das atividades nos restaurantes, academias, cultos religiosos, festas e passeios turísticos tensionou as relações, “*os donos de lanchonetes, bares e conveniências foram o que mais resistiram*” (SMS2). As atividades nas escolas estaduais foram as primeiras a serem suspensas: “*o estado decretou*” (PESF14), mas a população apresentou resistência. As rondas sanitárias instaladas para orientar e verificar o cumprimento das medidas de contingenciamento, o toque de recolher, o uso de máscaras, o distanciamento social e a proibição de aglomerações também não foram aceitas. Para cumpri-las, e sem intenção de punir, a polícia apoiou os profissionais na fiscalização: “*caso precisasse, acionavam a polícia militar*” (SMS2).

Nos municípios rurais remotos, os povos tradicionais tiveram prioridade para a vacina da COVID-19. Foram estabelecidos locais de vacinação com horários estendidos, incluindo finais de semana, e os vacinadores visitaram os estabelecimentos: “*passamos pelos comércios*” (SMS2); mas, nem todos aderiram: “*quem não tomou é porque não quis*” (PESF/AB3).

Durante a pandemia, os gestores e profissionais buscaram parcerias e se empenharam para o desenvolvimento das atividades. O sistema de saúde foi reorganizado, os fluxos da rede de atenção foram redefinidos e os consórcios ampliaram as cotas de serviços. Entrevistados afirmaram que os entes federativos cumpriram seu papel na transferência de recursos financeiros em suficiência e no tempo adequado, mas expressaram preocupações com o acesso aos serviços de saúde em tempo hábil, atribuído, em parte, à capacidade instalada insuficiente, que dificultou a regulação, especialmente para casos de maior gravidade.

### Condução da vigilância em saúde no municípios rurais remotos: percepções dos acometidos pela COVID-19

Na percepção dos usuários entrevistados “*sempre vai haver alguma falha*” (Usu16.1). Apesar disso, reconheceram o empenho dos profissionais e gestores no desenvolvimento das ações de vigilância em saúde nos municípios rurais remotos: “*botou barreira, não entrava ninguém, carros eram lavados, nas lojas passavam produto, o combate foi bem*” (Usu14.1); “*não posso falar mal, quando descobriu, trabalhou mesmo*” (Usu14.3); “*trabalharam incansavelmente*” (Usu10.2); “*cada ação foi importante, mas a maior foi o isolamento*” (Usu11.1); “*quem não se cuidou realmente não quis, álcool com vontade, fizemos máscara, distribuímos*” (Usu5.2).

Dos entrevistados que adoeceram, a maioria reconheceu a gravidade da situação, mas não mencionou o autocuidado para evitar o risco. Segundo eles, os decretos faziam exigências, nem sempre por eles cumpridas: “*sem máscara em mercados não pode entrar, mas eu confesso que era bem relaxado*” (Usu16.2). A comunicação intensiva sobre os riscos e a gravidade da situação geraram desconforto - “*encheram um bocado de vez, porque eu ficava sem máscara*” (Usu16.1) -, mas não influenciaram na busca pelo serviço, mesmo entre aqueles que apresentavam sintomas: “*não sentia febre, aí um dia fiquei ruim*” (Usu14.2); “*fui na farmácia*” (Usu5.5); “*antes de diagnosticar COVID não sentia mais gosto, nem cheiro, estava com sintomas*” (Usu13.1).

Apesar de reconhecerem o empenho da gestão e dos profissionais, chama atenção a baixa adesão às medidas de contingenciamento da COVID-19, como o uso de máscara e o isolamento social: “*estava tomando cerveja e já estava com ela* [COVID-19]*. Eu tomava no mesmo copo dele*” (Usu10.4); “*estava na internet, televisão, assistindo direto, estava crescendo o índice de COVID*” (Usu11.4); “*às vezes, usava máscara*” (Usu16.2); “*acho que peguei no contato. Eu me sentia muito perturbado com a máscara, foi esse relaxamento de não ter usado*” (Usu11.4). Esses comportamentos, e o medo de buscar atendimento médico, são fatores que podem ter contribuído para a propagação do vírus e o agravamento dos casos.

Durante o adoecimento, afirmaram ter recebido orientações e visitas peridomiciliares de médicos, enfermeiros e ACS. Esses profissionais monitoraram os casos e forneceram suporte necessário para o tratamento e recuperação: “*a equipe ligava, falava por chamada de vídeo, acompanhava, diariamente*” (Usu8.2). Alguns afirmaram que usaram máscaras, usaram álcool em gel, e “*receberam medicação, oxímetro*” (Usu8.2) e termômetros.

O comportamento e a percepção de risco variaram entre os indivíduos que adoeceram, mas os depoimentos indicaram resistências às medidas de contingenciamento e podem ter contribuído para disseminar o vírus.

**Figura 2:**
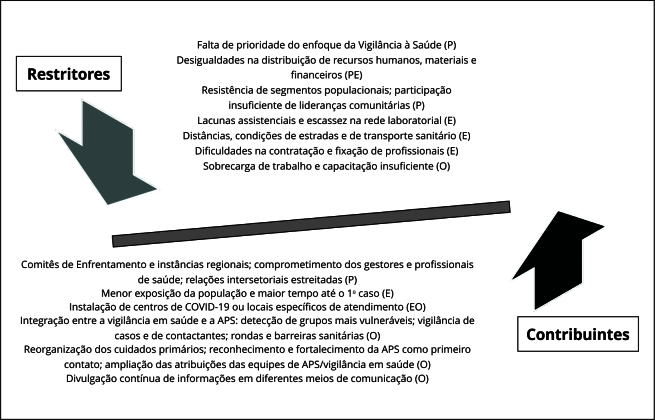
Fatores que contribuíram ou restringiram o enfrentamento da pandemia por parte dos municípios rurais remotos brasileiros (2021-2022).

A Figura 2 ilustra sinteticamente os principais fatores condicionantes do enfrentamento da pandemia. O Quadro 2 apresenta excertos de falas de entrevistados segundo as categorias estudadas.

APS: atenção primária à saúde. Nota: dimensões - E: Estrutura; O: Organização; P: Política.

**Quadro 2:** Relatos dos entrevistados, segundo condicionantes e categorias de análise. Municípios rurais remotos, Brasil, 2021-2022.

CONDICIONANTES	CATEGORIAS DE ANÁLISE	RELATOS DOS ATORES (GESTORES E PROFISSIONAIS DE SAÚDE)	PERCEPÇÃO DOS USUÁRIOS
Político	Condução política e o poder decisório quanto ao: Plano de contingência Comitê de Enfrentamento, medidas de contingenciamento, medidas consideradas mais importantes na percepção de atores Intersetorialidade - no trabalho de contingenciamento	“*...cada uma vinha com uma ideia diferente para ajudar o outro com mais dificuldade e aí foi muito importante*” (SMS4) “*Eu acredito que fez o que tinha que ser feito, incentivava o uso de máscara, fez algumas restrições em questão de aglomeração,* (...) *trabalharam incansavelmente para tentar diminuir a contaminação, pra mim foi ponto positivo, o trabalho que foi exercido*” (SMS10) “*Nós já fomos montando nossa equipe de enfrentamento, duas vezes por semana a gente se reunia, conversava, lia jornal e matéria sobre o COVID, era a equipe toda, equipe de enfrentamento trabalhava dia e noite*” (SMS5) “*Para sair e para entrar a pessoa tinha que vir aqui na Secretaria da Saúde, tinha que contar sua história e a gente avaliava se ia liberar ou não, infelizmente, muita gente ficou brava com a gente nesse período*” (SMS10)	“*...muito importante, as máscaras, não deixou faltar* [SMS]*, tinha, os cartazes, indicando para as pessoas, os cuidados que deveria tomar, não faltava álcool em canto nenhum. E assim, afastou, tirou as festas, tirou, isolou*” (Usu5.1) “*O que podia ter sido feito, fizeram, né*” (Usu11.2) “*As pessoas que não tiveram consciência, mas o trabalho deles, nas escolas sabe, eu falo, as medidas que eles tomaram, a gente seguia. era muita cobrança, ó, não pode deixar entrar mais de 5 pessoas. Ser obrigatório o uso de máscara, álcool em gel. Então eles exigiam isso, só que assim, o cidadão as vezes não colaborava*” (Usu11.2) “*Eu acho que combateu bem, no começo, aquele cuidado, botou barreira, não entrava ninguém de fora, o carro que entrava era tudo lavado já lá fora. Acho que, foi bem o combate foi bem*” (Usu14.1) “*Cada ação foi importante, mas a maior foi o isolamento. No início foi, que a COVID demorou a chegar aqui porque o município realmente*” (Usu11.1) “*É, fechou* [as entradas] *e ficava monitorando, demorou a chegar à COVID aqui no município*” (Usu11.1) “*Quem não se cuidou realmente não quis, máscara tinha com vontade, álcool com vontade, pra tudo quanto é lado*” (Usu5) “*Às vezes até polícia andava por aí, sem máscara tinha multa se te pegassem sem máscara. Chamou a responsabilidade da população. Tem as pessoas que não dava ouvido, né, não dá atenção, mas eu acredito que eles cumpriram o papel como deveria ter feito*” (Usu16.2) “*A própria população acaba passando por cima, das leis, das exigências, sanitárias*” (Usu11.4) “*Entendi. Aceitar, né? É, principalmente os mais jovens no início né. Mas depois que foi acomodando, foi caindo na real né, a preocupação estava aí pra todo mundo*” (Usu11.4) “*Foi a vacina, todo mundo vai tomar, que eles providenciam, né. E quem não toma mesmo é porque não quer*” (Usu8.7) “*Esse cuidado a prefeitura teve com os pacientes infectados, que ficaram em casa, a equipe ligava, falava com eles por vídeo, acompanhava, diariamente eu acho que fizeram o possível mesmo que não foi fácil*” (Usu8.2)
Estrutural	Mudança estrutural e adaptações na estrutura física existente Capacidade instalada laboratorial Aquisição dos insumos específicos e equipamentos de apoio laboratorial e de proteção individual (máscaras, sanitizantes, oxímetros, O_2_, termômetro) RT-PCR e TC Disponibilidade de testes rápidos Vacinação - rede de frio Equipe de vigilância Disponibilidade de transporte, envio de amostras laboratoriais e encaminhamentos dos acometidos Estrutura de TIC	“*Um verdadeiro transtorno, a gente mora muito longe, quatrocentos e poucos quilômetros, tinha que estar indo praticamente quase todos os dias, para levar esses swab*” (SMS2) “*A tomografia aqui mais próxima era, 90 quilômetros, então era ambulância indo duas, três vezes para lá e tinha que levar só um paciente*” (AS4) “*Tinha as ACS que a todo tempo monitoravam as microáreas, se precisasse de atendimento específico elas ligavam, a enfermeira marcava o horário e o paciente ia até lá, era assim que funcionava*” (AS4) “*Zonas rurais tem acesso à internet, então lançava, vídeos educativos com o pessoal da vigilância, com enfermeiro, com nutricionista*” (SMS14) “*A gente não passou nenhum período sem, mas tinha preocupação a gente fez o pedido e demorava a chegar, quebras de contrato, antes da pandemia era um valor, quando chegou a pandemia os valores triplicaram, quadriplicaram, e as empresas não entregavam*” (PESF/VS4)	
Organizacional	Acompanhamento e atendimento presencial e remoto Separação do acesso para rotina e sintomáticos respiratórios Definição de equipes específicas para acompanhamento dos sintomáticos Realocação de profissionais na equipe de vigilância Ampliação das atribuições dos profissionais da APS para vigilância em saúde Disponibilidade de protocolos organizacionais Fluxos e reorganização dos serviços de atenção à saúde para diagnóstico e monitoramento Fluxos e reorganização dos serviços de apoio laboratorial local e regional Monitoramento dos sinais clínicos dos sintomáticos em isolamento domiciliar Notificação de casos pelas equipes Busca ativa de sintomáticos e de contatos Comunicação de risco e educação em saúde	“*Aqui, tem a unidade básica de saúde, o PSF, e do outro lado é esse o centro da COVID, é separado, até a equipe é separada*” (AB2) “*As pessoas mais distantes conseguem ouvir, eram dadas entrevistas, os meios de proteção individual, aglomeração, distanciamento social, foram muito abordadas*” (AB10) “*A gente viu realmente o que era trabalhar em equipe com a COVID, um ajudava o outro, não tinha essa história, sou fisioterapeuta eu não vou fazer isso aqui, sou psicóloga não vou estar aqui, todo mundo se envolveu e cada um fez um pedacinho*” (SMS14) “*No início além da demanda da atenção básica, tinha que dar assistência as pessoas com sintomas de COVID, coletar material, monitorar no início foi bem sobrecarregado*” (PESF16) “*A UBS fluvial era a barreira sanitária, a equipe de saúde nessa entrada do município, fazia as orientações para quem tentasse entrar*” (SMS10) “*Nas entradas da cidade, atuavam os ACS, a vigilância, a polícia militar, tinha o conhecimento de quem entrava e saía do município, e fazia as orientações*” (AB, 13) “*A suspensão das aulas, festas, bares, teve supervisão da vigilância sanitária, durante o dia e a noite; as feiras tiveram horários e dias específicos para abrir, teve toque de recolher, as igrejas tiveram que fechar, as escolas, alguns comércios que não eram essenciais também foram fechados*” (AB10) “*Seguimos a orientação, reduzimos o fluxo da unidade, os serviços foram suspensos e depois de um certo período nós entendemos que teríamos que retomar, tivemos que reavaliar todo o funcionamento da unidade*” (AB3) “*As pessoas esperavam todos os dias as oito horas da noite a publicação do doutor* [prefeito] *falando da COVID, começavam falando da COVID, como evitar, aí entrava número de casos, quantos hospitalizados*” (PESF8) “*Deixou a desejar a não implantação da barreira sanitária, porque aqui tem um fluxo muito grande de pessoas de outros municípios vem para os ranchos e passa, no comércio local, as vezes sem uso adequado da máscara*” (PESF/AB3) “*Pensamos em fazer* [barreira sanitária]*, mas ouvindo experiências de municípios a gente preferiu não fazer as vezes se a pessoa está com febre, se tomou uma dipirona a febre não está, então não é uma coisa que teve êxito então eu não adotei*” (SMS2) “*Festas clandestinas, denúncias, foi difícil, bem complicado, o Ministério Público ajudou bastante, foi bem parceiro*” (SMS2)	

ACS: agente comunitário de saúde; APS: atenção primária à saúde; PSF: Programa Saúde da Família; RT-PCR: reação em cadeia da polimerase com transcrição reversa; SMS: Secretaria Municipal de Saúde; TC: tomografia computadorizada; TIC: tecnologia de informação e comunicação; UBS: unidade básica de saúde.

Fonte: elaboração própria.

Nota: atores - AB (coordenador de atenção básica); AS (assistente social); PESF (profissional da Estratégia Saúde da Família); SMS (Secretário Municipal de Saúde); Usu (usuário dos serviços de saúde); VS (coordenador da vigilância em saúde). Os números referem-se aos municípios rurais remotos (variam de 1 a 16, admitindo mais de um usuário por município).

## Discussão

Na pandemia da COVID-19, as desigualdades socioespaciais dos municípios rurais remotos geraram mais desafios e dificuldades para reorganizar, operacionalizar e acessar a rede de apoio à vigilância em saúde ^12^ ^,^ ^17^ ^,^ ^18^ ^,^ ^19^, e exigiram gestão de riscos e governança intra e intergovernamental robustas ^25^.

No Brasil, a exposição aos riscos e agravos durante a pandemia tornou-se mais evidente nos municípios rurais remotos. As unidades de saúde reorganizaram a rotina, adaptaram seus fluxos e atenderam de forma diferenciada os inscritos nos programas básicos de saúde ^12^ ^,^ ^26^. As ações foram intensificadas, os profissionais da vigilância em saúde realizaram a entrega domiciliar de medicamentos, mantiveram contato telefônico com a população e buscaram parcerias com outros setores. Esses municípios obtiveram êxitos, mas enfrentaram dificuldades pelas singularidades do território e situações de desigualdades que os caracterizam ^12^ ^,^ ^19^.

Os principais fatores que condicionaram a organização e operacionalização da vigilância em saúde foram: as lacunas assistenciais da rede de atenção pública e privada, local e regional; a escassez e desigualdades na disponibilidade e distribuição de equipamentos, serviços e profissionais na rede locorregional de apoio laboratorial; a distância até os grandes centros; as condições das estradas; e as dificuldades para o transporte sanitário adequado ^12^ ^,^ ^19^. Essas condições específicas e desfavoráveis dos territórios rurais, antes e durante a pandemia ^12^ ^,^ ^20^ ^,^ ^25^ ^,^ ^27^, contribuíram para expor a população a mais riscos e maior insegurança, além de aumentar os custos municipais.

Os gestores se empenharam, tiveram iniciativa para organizar e integrar a vigilância em saúde à APS, receberam transferência de recursos financeiros, mas a estruturação da rede de atenção locorregional requer prioridade no financiamento e estabelecimento de pactos interfederativos ^5^ ^,^ ^18^ ^,^ ^20^ ^,^ ^28^.

Os indicadores sociodemográficos, econômicos e de infraestrutura local indicam que as desigualdades ^18^ ^,^ ^19^ que influenciaram e condicionaram a operacionalização da vigilância em saúde e o cuidado integral da população residente nos municípios rurais remotos ^17^ ^,^ ^18^ ^,^ ^19^ ^,^ ^29^ já existiam antes da pandemia, sendo significativamente maiores em comparação com as populações urbanas ^30^. Além disso, nos municípios rurais remotos existem fragilidades nas relações intergovernamentais ^20^ ^,^ ^28^ ^,^ ^31^ que podem resultar em lacunas na prestação de serviços de saúde, financiamento inadequado e alocação insuficiente de recursos para estruturar os serviços de saúde ^20^.

A remoticidade e as decisões dos comitês sobre medidas antecipadas de contingenciamento e vigilância, a suspensão das atividades essenciais e não essenciais, a definição de locais distintos para triagem de fluxos de atenção para sintomáticos respiratórios e casos suspeitos foram fundamentais para controlar e detectar casos e contatos, e contribuíram para retardar a ocorrência do primeiro caso, superando o tempo registrado nos respectivos estados. Comparativamente aos grandes centros urbanos, os municípios rurais remotos foram favorecidos pela menor exposição da população a aglomerações ^5^ ^,^ ^29^.

A cooperação técnica das instâncias regionais/SES estimulou e orientou os gestores e comitês de enfrentamento a tomarem decisões. Os gestores foram ágeis na instalação dos centros de COVID-19 ou locais específicos para atender as pessoas com sintomas respiratórios. Em alguns municípios rurais remotos, a rede de apoio laboratorial e logística foi ampliada e readequada, e foram adquiridos equipamentos e insumos, mas a demora na obtenção dos resultados de exames, as dificuldades na regulação e a inexperiência das equipes no manejo da COVID-19 afetaram a vigilância, as notificações, o monitoramento e o tratamento de casos.

As transferências de recursos financeiros governamentais ocorreram, mas os investimentos não advieram em todos os municípios rurais remotos, não foram imediatos e não supriram as necessidades. A crise gerada pela COVID-19 ressaltou as dificuldades para acessar os serviços em tempo oportuno e a necessidade de intervenção interfederativa na rede de atenção, com investimentos públicos em curto, médio e longo prazos ^18^ ^,^ ^28^.

O empenho para desenvolver as atividades de vigilância em saúde integradas à APS contou com a dedicação dos profissionais das SMS e de outras secretarias, realocados para realizar as atividades ampliadas de vigilância. Os processos de trabalho foram ampliados e potencializados para detectar e identificar grupos vulneráveis ^32^, manter o cuidado para acolher as demandas e oferecer segurança tanto para o trabalhador como para o usuário em todos os níveis do sistema de saúde ^20^ ^,^ ^33^ ^,^ ^34^, mas geraram sobrecarga de trabalho.

As ações de vigilância em saúde e a divulgação sobre a situação pandêmica nos municípios rurais remotos podem ter contribuído para conter a disseminação do vírus ^26^ e obter o reconhecimento da população quanto aos esforços dos gestores e profissionais na condução. No entanto, as resistências, os diferentes comportamentos e percepções da população quanto aos riscos de adoecer podem indicar que as orientações e comunicações não foram abrangentes e inclusivas o suficiente. A implementação da vigilância em saúde integrada à APS é um desafio, requer investimentos, capacitações dos profissionais e reflexões que abarquem a sua concepção integral e participativa ^35^.

As decisões sob a governabilidade dos municípios rurais remotos foram tomadas, as atividades reorganizadas para atender às necessidades da população, mas as funções intergestores não estavam claras quanto à operacionalização da rede de atenção intermunicipal. A cooperação intergestores e a integração dos diferentes níveis de serviços de saúde são fundamentais para melhorar a eficiência, a equidade e a qualidade do atendimento. Estudos mostram que a liderança e a capacidade de negociação foram pontos fortes da gestão e governança no enfrentamento da crise na China, Alemanha e Espanha. Em contraste, no Brasil, esses aspectos foram pontos fracos, indicando a necessidade de arranjos interfederativos para reorientar o sistema de saúde e reduzir as lacunas assistenciais ^20^ ^,^ ^36^.

Os resultados deste estudo de casos múltiplos não garantem a representatividade do conjunto dos municípios rurais remotos, portanto, não podem ser generalizados; mas explicitam os processos vivenciados para reorganizar o sistema local de saúde. Diferentes situações mostraram que o empenho dos gestores e profissionais foram determinantes na utilização dos recursos e meios disponíveis para realizar a vigilância em saúde, alcançar a população da sede e das comunidades rurais ^6^, e favorecer a coesão da comunidade, apesar das limitações descritas. As melhores respostas locais foram as organizacionais, frente às dificuldades políticas e estruturais existentes. As diferentes perspectivas discutidas contribuem para o debate sobre as possibilidades e limites ^35^ da implementação dessas práticas para além da pandemia.

## Considerações finais

Durante a pandemia da COVID-19, os municípios rurais remotos enfrentaram dificuldades para garantir a operacionalização da vigilância em saúde e o acesso dos sintomáticos respiratórios aos serviços de saúde. Houve a necessidade de reorganizar a rede de atenção à saúde, obter cooperação técnica, realizar a governança intergestores e buscar alternativas para operacionalizar o sistema locorregional de saúde.

Esse período de crise sanitária exigiu a junção de esforços dos diferentes atores e a articulação entre ações de prevenção e controle de riscos à saúde. Os municípios rurais remotos demonstraram suas potencialidades em cumprir a Política Nacional de Vigilância em Saúde. O processo de trabalho imposto pela pandemia, desde o diagnóstico clínico até o acompanhamento das populações mais vulneráveis, indicou uma promissora implementação dos atributos comunitários e territoriais da Estratégia Saúde da Família nos municípios rurais remotos.

Os condicionantes políticos, estruturais e organizacionais confirmaram a insuficiência de serviços local e de referência, a baixa capacitação dos profissionais e as fragilidades na comunicação entre os níveis de governo. Nesse cenário pandêmico, os entrevistados consideraram que os entes federativos cumpriram seu papel, transferindo recursos financeiros e ou destinando localmente em suficiência e no tempo adequado.

Embora não consensual, as críticas foram direcionadas ao governo estadual pela insuficiência da rede de atenção regionalizada que reconhecem preceder a pandemia, e ao Governo Federal pelo atraso no envio das vacinas. Os gestores locais se esforçaram para conter os riscos, mas as vulnerabilidades locais confirmam que esses territórios demandam políticas públicas equitativas, coordenação intergovernamental proativa, investimentos em infraestrutura e redistribuição de profissionais.

O estudo mostrou que, na condução da pandemia da COVID-19, com limitações, os municípios rurais remotos desempenharam suas funções, estabeleceram arranjos e relações intersetoriais locais, integraram as atividades da vigilância em saúde à APS, reorganizaram os processos de trabalho e a comunicação com a população. Entretanto, as atividades que exigiram articulação interfederativa para fortalecer a rede assistencial, o apoio laboratorial e o sistema logístico foram insuficientes, sendo estes os principais obstáculos para o cuidado individual e coletivo.

A responsabilidade compartilhada interfederativa pode aprimorar as políticas de saúde e garantir a inclusão dos municípios rurais remotos na agenda de prioridades. A crise da pandemia revelou que as práticas de vigilância em saúde integrada à APS são necessárias não apenas nas crises sanitárias, mas na construção de um sistema de saúde mais resiliente e sustentável, que cumpra os princípios da integralidade, intersetorialidade e participação social.
